# Perceived Perioperative Competence, Self-Efficacy, and Job Burnout Among Chinese Operating Room Nurses: A Cross-Sectional Study

**DOI:** 10.3390/healthcare13243218

**Published:** 2025-12-09

**Authors:** Yaqin Li, Weihao Kong, Lingli Li

**Affiliations:** 1 Department of Nursing, West China Hospital/West China School of Nursing, Sichuan University, Chengdu 610041, China; yaqin0809.li@connect.polyu.hk (Y.L.); 2022151620537@stu.scu.edu.cn (W.K.); 2 Nursing Key Laboratory of Sichuan Province, Chengdu 610041, China

**Keywords:** perceived perioperative competence, operating room nurses, current status, associated factors, self-efficacy, job burnout, mediating effects

## Abstract

**Background**: To ensure smooth surgical procedures, patient safety, and quality of perioperative care, perceived perioperative competence (PPC) is a daily and urgent requirement for operating room (OR) nurses. Understanding the status of PPC among OR nurses and its associated factors is essential for OR nurses/people in charge/researchers to pinpoint weaknesses and formulate interventions. Therefore, we aim to investigate the status of PPC and its associated factors. Furthermore, we explore the relationship between PPC, self-efficacy, and job burnout among OR nurses. **Methods**: Tertiary-A hospitals in various districts of Chengdu, China, were recruited using a stratified convenience sampling method. A cross-sectional survey was then administered to OR nurses in the selected hospitals. Data analysis included descriptive analysis, T-tests, one-way analysis of variance (ANOVA), multiple linear regression analysis, correlation analysis, and mediation analysis. **Results**: A survey of 640 OR nurses across 18 hospitals (with a 98.00% valid response rate) revealed an average PPC score of 3.66 ± 1.12/124.55 ± 26.54. Marital status, OR specialty education, and age significantly influenced PPC levels (*p* < 0.05). Self-efficacy was positively correlated with PPC, while job burnout was negatively correlated with PPC. Self-efficacy fully mediated the relationship between these two variables. **Conclusions**: The performances of PPC among Chinese OR nurses were acceptable. Marital status, OR specialty education, and age significantly influenced PPC levels. Self-efficacy fully mediates the relationship between job burnout and PPC.

## 1. Introduction

The operating room (OR) is a highly stressful and demanding environment [1] where staff must maintain focus, use advanced skills, work as a team [2,3,4,5], make quick decisions [6,7], manage complex equipment [8], and endure long surgeries [9], which are physically and mentally challenging for OR staff. OR nurses are one of the most crucial groups, as the proportion of OR nurses comprises over 50% of the OR team, making them integral to the operating team’s functionality [10,11]. Additionally, OR nurses play a vital role in ensuring patient’s safety and in enhancing organizational efficiency [12]. Ensuring the quality of perioperative care and perceived perioperative competence (PPC) is a daily and urgent requirement for OR nurses [13]. PPC refers to comprehensive abilities during perioperative period, which can fall into two categories, technical and non-technical competencies [14,15,16,17,18], including knowledge, skills, motivation, and traits essential for OR nurses. On one hand, technical competencies refer to abilities/skills encompassing aseptic skills such as the preparation/management of surgical instruments, and the monitoring/management of technical equipment in OR units [16,17]. OR nurses operate under high-pressure conditions, with a critical emphasis on maintaining strict aseptic techniques and quality standards [19]. On the other hand, non-technical competencies involve clinical judgment, leadership, decision-making skills, information literacy/management skills, holistic patient care, practical communication skills, empathy, and teamwork [14,15]. To guarantee patients receive thorough and top-notch care during surgery, OR nurses must adeptly blend technical and non-technical competencies [15].

Regular assessment of the current status of PPC of OR nurses and its associated factors contribute to the provision of ethical, safe nursing care with great quality [20]. The Perceived Perioperative Competence Scale–Revised (PPCS-R) [21], developed in 2012, with Cronbach’s alpha of the whole scale of 0.96, stands as the first rigorously developed/validated measurement tool of PPC among OR nurses. This tool was proved highly effective in measuring PPC in OR nurses [13], offering comprehensive content [13,21,22], and used for validation across multiple countries (e.g., China, U.S., Sweden) along with satisfactory psychometric properties [13,22,23]. Various studies worldwide have assessed the current PPC status and its associated factors using this reliable scale in their validated version. For instance, studies from south Korea investigating PPC among OR nurses (N = 318, 3.78/5.00) [24], along with studies from Sweden (N = 505, 38.40/45.00) [25], found that OR nurses generally had high levels of PPC. Research has also explored the associated factors impacts on the PPC of OR nurses, including educational level [26,27,28], OR work experience [26,27,28], gender [25], recency of training [27], and clinical ladder programs [24], which have been proven to significantly influence OR nurses’ PPC levels. However, our review of the literature revealed that few studies have measured the current status of Chinese OR nurses and its associated factors using reliable and validated tools.

Self-efficacy refers to an individual’s perception of their ability to perform a task successfully [29]. A strong sense of self-efficacy empowers individuals to exercise autonomy in their decision-making, as confidence in one’s abilities reduces reliance on external guidance and fosters proactive and independent problem-solving [30,31]. Some research explored the relationship between nurses’ PPC/competence and self-efficacy, and most of the findings suggest a positive relationship between nurses’ PPC/competence and self-efficacy. For instance, one study aiming to develop a simplified version of the Australian PPCS-R for 485 OR nurses revealed a positive correlation between nurses’ PPC levels and their overall self-efficacy [27]. Another study involving 119 OR nurses in Taiwan on the relationship between personality traits, self-efficacy, and PPC found a significant positive correlation between self-efficacy and PPC (r = 0.54, *p* < 0.001) [28].

Job burnout, encompassing emotional exhaustion and a diminished sense of career achievement, is a widespread phenomenon in modern workplaces [32]. Research consistently demonstrates an inverse relationship between job burnout and competence. A study of 809 nurses found that job burnout adversely affects their professional competence [33]. Similarly, research involving 385 doctors identified a negative correlation between burnout and work competence [34]. Lastly, a meta-analysis investigated the relationship between self-efficacy and burnout (57 studies, N = 22,773), revealing a moderate negative correlation between self-efficacy and burnout [35]. These three components, namely, PPC, job burnout, and self-efficacy, establish a solid triangular closed loop, each influencing the other factors. Additionally, from the review of the literature above regarding the relationship among PPC, job burnout, and self-efficacy, it is not hard to hypothesize that self-efficacy may potentially serve as a mediating factor that impacts people’s PPC/competence and level of job burnout. However, the review of the literature conducted by us reveals that there is no research or theoretical foundation to support the relationship among these three components. The relationship among self-efficacy, job burnout, and OR nurses’ PPC remains unexplored and warrants investigation.

Hence, this study aims to assess the status of PPC among Chinese OR nurses alongside its associated factors. Additionally, it is the first study that has investigated the interconnections among OR nurses’ PPC, self-efficacy, and job burnout. To analyze the complex relationships among these three variables, we employed structural equation modeling. This method is uniquely suited to test both direct and indirect effects via path analysis while modeling latent variables, thereby providing a robust framework for uncovering their underlying mechanisms. By examining the relationship among these three variables within China’s unique context, our study not only contributes to the local understanding but also offers important insights for international literature by demonstrating how self-efficacy influences job burnout and the PPC of OR nurses.

## 2. Materials and Methods

This study adhered to the STROBE checklist for cross-sectional studies [36].

### 2.1. Design, Study Setting, and Data Collecting

Chinese hospitals are classified into three levels (e.g., primary, secondary, tertiary), each further divided into A, B, and C grades. Tertiary-A hospitals, which represent the highest tier in China [37,38], were sampled from Chengdu, a city comprising 12 municipal districts [39]. A stratified convenience sampling method was employed in this study to guarantee the selection of at least one tertiary-A hospital from each district. The procedures were as follows: A list of tertiary-A hospitals in Chengdu from the Chengdu Municipal Government website was obtained. The hospitals were contacted (e.g., by telephone, email, or reaching out to acquaintances) one by one to acquire their OR’s contact information. If we were unable to find contact information for a hospital, we moved on to the next one. If we obtained informed consent from at least one OR within a district, we then proceeded to the next district.

This study was cross-sectional, and the data collection was conducted from January 2023 to June 2023. The sample size for this study was determined using the formula for estimating the population rate in cross-sectional surveys [40]. Among the three key variables in this study (PPC, self-efficacy, and job burnout), only job burnout has a prevalence rate. Consequently, the sample size calculation for this study was based on the prevalence rate of job burnout. Calculation parameters were set as follows: 95% confidence level (Z = 1.96), with a permissible error of 5%. The anticipated prevalence rate was determined by referencing Lin Shuqiu et al.’s meta-analysis (20 studies, N = 4061) on the incidence of job burnout among OR nurses in China [41], revealing that severe low personal accomplishment was the most prevalent issue among OR nurses, reaching 47.3%. Based on this conservative estimate, the minimum effective sample size was calculated to be 383 participants. Considering an approximate 15% non-response rate, the final objective for this study is to recruit at least 450 OR nurses.

Data collecting processes were as follows: after contacting the head nurse in OR and receiving their support, electronic questionnaires were distributed through their online work group (e.g., via WeChat App version 8.0.65 or QICQ App version 9.9.18-36553) or distributed as paper questionnaires during their department meetings on-site. OR nurses were provided with instructions on the completion of this questionnaire and an informed consent form on the cover page to ensure clarity. Researchers promptly addressed any ambiguities or confusion, both online and on-site. Once all electronic and paper questionnaires were completed, quality check was conducted right after their completion to ensure appropriate quality of responses. The electronic questionnaires could not be submitted if the responses were incomplete, the completion time was too short (less than 200 s), or all questions had the same answer selected (e.g., all “totally agree”). For paper questionnaires, our researchers immediately reviewed them for completeness and quality checked after they were completed by OR nurses. If any unclear responses were identified, they were promptly verified with the respondents.

### 2.2. Inclusion and Exclusion Criteria

Inclusion criteria of OR nurses were as follows: (1) nurses registered in mainland China; (2) working full time in the OR in a tertiary-A hospital in Chengdu; (3) engaged in daily OR work; (4) aged 18 and above; (5) provided informed consent. Exclusion criteria encompassed (1) trainee nurses and (2) nurses with less than one month of work experience with potential risk of incomplete understanding of OR nursing routines.

### 2.3. Instruments

The instruments comprised five elements: an introduction of the study (e.g., the study’s purpose, survey procedures, participant’s right to withdraw anytime) along with an informed consent form, a demographic questionnaire, the 34-item Chinese Perceived Perioperative Competence Scale-Revised (C-PPCS-R34) [13], the Chinese version of the General Self-Efficacy Scale (GSES-C) [42], and the Chinese version of the Maslach Burnout Inventory-Human Services Survey (MBI-HSS) [43]. Demographic information encompassed general background details about OR nurses, including age, gender, and marital status. We obtained permissions for the use of the following three Chinese version scales from their copyright owner.

#### 2.3.1. The 34-Item Chinese Perceived Perioperative Competence Scale-Revised (C-PPCS-R34)

Yu et al. [13] introduced and culturally adapted the original PPCS-R scale [21] into Chinese culture based on a population of 480 Chinese OR nurses. A total of 34 items comprise this scale. The C-PPCS-R34 demonstrated satisfactory reliability and validity, including a content validity index of 0.875 and Cronbach’s alpha of 0.787 for the total scale, while each dimension ranged from 0.792 to 0.949. Additionally, confirmatory factor analysis confirmed a six-dimension model including foundational knowledge and skills, leadership, collaboration, proficiency, empathy, and professional development, explaining 68.62% of the variance. Response scales used five-point Likert scales, ranging from never (one point) to always (five points). The total score for this scale was 170: the higher the total scoring, the better the PPC.

#### 2.3.2. The Chinese Version of the General Self-Efficacy Scale (GSES-C)

The Chinese version of the General Self-Efficacy Scale, adapted by Zhang and Schwarzer (the original GSES developer) in 1995 [42], consists of 10 items in a mono-dimensional structure, with a total Cronbach’s alpha value of 0.910. The response scale applied four-point Likert scales, ranging from completely incorrect (one point) to completely correct (four points). The scale’s total score ranged from 10 to 40, with higher scores indicating a greater perception of self-ability. Generally, scores between 10 and 20 were considered low-level, self-efficacy scores between 21 and 30 were considered average, and scores between 31 and 40 were considered high.

#### 2.3.3. The Chinese Version of the Maslach Burnout Inventory-Human Services Survey (MBI-HSS)

Developed by Maslach and Jackson in 1981 [44], the original Maslach Burnout Inventory is a scale globally utilized to measure job burnout. The Chinese version of the MBI-HSS was translated and culturally adapted by Xiaomei Li and Yanjun Liu [43] in a study conducted on 239 nurses from four hospitals in Xi’an city, Mainland China, in 2000. The confirmatory factor analysis of the Chinese version of the MBI-HSS scale revealed a three-dimensional structure, with good structural reliability: the X^2^/df was 1.928, with a significance level of *p* < 0.001; the Root Mean Square Error of Approximation (RMSEA) index was 0.048; the Comparative Fit Index (CFI) was 0.914; and the Tucker–Lewis Index (TLI) was 0.935 [45]. The MBI-HSS has 22 items and a total Cronbach’s alpha of 0.93 [43]. Cronbach’s alpha for the three dimensions in the MBI-HSS was (0.91) for emotional exhaustion, (0.81) for depersonalization, and (0.84) for personal accomplishment [43]. A seven-point Likert scale ranging from never (zero point) to always (six points) was used in the questionnaire as the response scale. Scores indicative of moderate or high burnout for each dimension were 19–26 or ≥27 for emotional exhaustion, 6–9 or ≥10 for depersonalization, and 34–39 or ≤33 for personal accomplishment.

#### 2.3.4. Data Analysis

Data analysis was conducted using SPSS 27.0 (subscriptions from Sichuan University) and Amos 28.0 (SPSS statistics, subscriptions from Sichuan University). No missing data were found in this survey. Descriptive analysis involved frequency, percentage, mean, and standard deviation (SD). T-tests and one-way analysis of variance (ANOVA) explored differences in PPC scores among OR nurses based on various demographic indicators. Correlation analysis (among PPC, self-efficacy, and job burnout) and multiple linear regression analyses (independent variables: self-efficacy and job burnout; dependent variable: PPC) were employed to analyze associated factors related to OR nurses’ PPC. Additionally, Amos 28.0 facilitated the construction of a structural equation model to test the hypothesized relationships between job burnout, self-efficacy, and PPC.

#### 2.3.5. Ethical Consideration

This study received approval from the Biomedical Ethics Review Committee of XX Hospital of XX in January 2023 (Approval Number: 2023–26). Clinical trial number: not applicable. An informed consent form outlined the study’s purpose and questionnaire completion procedures; precautions were provided before the survey to all participants, ensuring anonymity and exclusive use of data for academic purposes. Respondents retained the right to withdraw from the study at any time.

## 3. Results

Eighteen hospitals from 12 districts in Chengdu were involved in this study, with at least one hospital chosen per district. Four districts involved more than two hospitals, Jinjiang district (N = 3), Wuhou (N = 3), Jinniu (N = 2), and Qingyang (N = 2). A total of 640 questionnaires were initially distributed. After excluding 13 questionnaires due to their short completion time and low quality of responses, 627 questionnaires passed the quality check, which met the estimated sample size, resulting in an effective questionnaire rate of 98.00%.

### 3.1. Demographic Information

Of the 627 individuals, 83.25% were female, 60.77% were married, and the mean age was (31.91 ± 7.31) years. Further details can be found in Table 1.

### 3.2. The Current Status of the OR Nurses’ PPC

The total score of C-PPCS-R34 was (3.66 ± 1.12/124.55 ± 26.54), in which sub-dimension empathy (3.93 ± 0.98/19.66 ± 4.45) scored the highest, and professional development scored the lowest (3.41 ± 1.09/17.05 ± 4.60).

### 3.3. Associated Factors of the PPC

Statistically significant associated factors were found among various demographic factors related to the PPC scores. The independent *t*-test analysis identified marital status (t = −6.6, *p* < 0.001) and obtained OR specialty education (t = 3.5, *p* < 0.001) as associated factors. Furthermore, ANOVA demonstrated that several other variables had a statistically significant impact on PPC scores. These included age (F = 25.022, *p* < 0.001), time spent working in the OR (F = 32.47, *p* < 0.001), employment type (F = 4.078, *p* = 0.017), monthly income (F = 7.393, *p* < 0.001), professional rank (F = 18.683, *p* < 0.001), and self-awareness of current physical condition (F = 13.288, *p* < 0.001). A comprehensive breakdown of these results is presented in Table 1.

### 3.4. Correlation Analysis

A statistically significant, moderately strong positive correlation between PPC and self-efficacy (r = 0.604, *p* < 0.001) was identified. In contrast, PPC demonstrated a significant inverse correlation with job burnout (r = −0.357, *p* < 0.001). Lastly, a statistically significant negative correlation between self-efficacy and job burnout was identified (r = −0.378, *p* < 0.001). More details can be found in Appendix A.

### 3.5. Multiple Linear Regression Analysis

The inclusion of self-efficacy and job burnout in multiple linear regression analysis yielded a significant regression equation (F = 194.233, *p* < 0.001). Both self-efficacy (β = 0.547, *p* < 0.001) and job burnout (β = −0.151, *p* < 0.001) demonstrated statistical significance on OR nurses’ PPC scores. Collectively, these variables explained 38.20% of the variation in OR nurses’ PPC scores. Further details can be found in Appendix B.

### 3.6. Mediation Analysis

The structural equation model was analyzed using AMOS 28.0. The resulting fit indices included χ^2^/df = 2.360, RMSEA = 0.047, Standardized Residual Mean Root (SRMR) = 0.093, Goodness-of-Fit Index (GFI) = 0.797, Normed Fit Index (NFI) = 0.863, Robust Fitting Index (RFI) = 0.855, Incremental Fit Index (IFI) = 0.916, TLI = 0.911, and CFI = 0.916. Although the SRMR, GFI, NFI, and RFI values were less ideal, the remaining indicators satisfied the standard thresholds. This pattern of results supports the conclusion that the model has an acceptable fit for conducting path analysis.

The analysis demonstrated that job burnout had a significant indirect effect on PPC through the mediating role of self-efficacy (indirect effect = −0.18, *p* = 0.020). In contrast, the direct effect of burnout on PPC was not statistically significant (direct effect = −0.07, *p* = 0.062). This pattern of results indicates that self-efficacy fully mediates the relationship between job burnout and professional practice competence. Further details are illustrated in Table 2.

## 4. Discussion

In this study, we included 18 tertiary-A hospitals from 12 districts in Chengdu using stratified convenience sampling, ensuring representation from each district. However, because stratified convenience sampling may limit the generalizability and representativeness of the included samples compared to the entire population of all OR nurses in China [46], the results of this study primarily reflect the reliability of the included nurses. Consequently, these findings should be extrapolated to the entire population with caution. We distributed 640 scales and successfully obtained responses for 627, achieving an impressive 98.00% response rate. For comparison, Brigid et al. (2023) developed the short-form version of the PPCS-R scale among 677 Australian OR nurses with an effective response rate of 71.64% (N = 485) [27], and Shannon et al. (2024) introduced and tested the psychometric properties of the PPCS-R scale among 1661 American OR nurses with 1581 valid questionnaires (95.18%) [22]. Our higher response rate highlights the effectiveness of our data collection process.

The participants were mostly female (83.25%), with over half (52.31%) aged 26 to 35 years, and 34.77% of the participants had accumulated over ten years of experience in the OR. The educational and professional background was notable, with 80.38% holding a bachelor’s degree or higher, and 80.22% achieving a senior registered nurse status or a higher professional rank. However, only 14.51% held tenured positions. Comparatively, in Brigid et al.’s study (2023) [27], the subjects were predominantly female, with an average age of 48.6 ± 10.8 years and an average OR work experience of 19.6 ± 11.7 years. Over 70.3% held a postgraduate certificate in perioperative nursing, 61.0% had postgraduate education or relevant training in perioperative nursing, and 30.1% had received more than three years of specialized perioperative training [27]. In Shannon et al.’s study (2024), 89.7% were female, 72.9% held professional certifications, 59.3% held bachelor’s degrees, and the average perioperative nursing experience was 17.8 ± 11.5 years [22]. Compared to previous studies, our participant demographics showed both similarities and differences. Our sample was younger than that in Brigid et al.’s study [27]. Furthermore, while the proportion of participants holding a bachelor’s degree was higher than in Shannon et al.’s study [22], the proportion with graduate-level education was lower than in Brigid et al.’s [27]. Finally, the percentage of individuals with over ten years of OR experience was lower than in both of the earlier studies.

The overall score for PPC in this study was (3.66 ± 1.12), indicating a moderate level on a five-point scale. When compared to the findings of the other studies, the PPC score in our study was comparatively lower than those reported in a Swedish study surveyed on their OR nurses and anesthetists using PPCS-R (3.96 ± 0.50, N = 505, and 3.96 ± 0.48, N = 528, respectively) [25]. However, our result was higher than the score reported by Yu et al. (3.28 ± 0.18) [13] for her adaptation study of the original PPCS-R scale into Chinese culture. General or professional-targeted education or training, along with relevant work experience, has been identified as having significant impacts on job competency or performance [47,48]. The higher proportion of participants holding bachelor’s or higher degrees in our study may explain why our PPC scores were higher than those reported in Yu et al.’s study [13]. However, compared with the Swedish cross-sectional survey study [25], our scores were lower, likely due to the older average age (48.8 ± 9.5 years) and greater perioperative experience (18.5 ± 10.9 years) among the OR nurse sample in the Swedish study [25].

The empathy category received the highest average rating (3.93 ± 0.98), in contrast to the leadership category, which had the lowest mean score of (3.26 ± 1.21). This disparity not only reflects differing developmental levels in emotional cognition and organizational management capabilities among nurses but also suggests potential structural biases within the current nursing educating system. Empathy, defined as an individual’s cognitive ability to understand others’ emotional states [49], plays a foundational role in nursing practice. Nurses’ strong performance in this dimension may be attributed to the curriculum design in China emphasizing nursing ethics, psychology, and professional responsibilities. This fosters the ability to consider patients’ perspectives, rapidly identify emotional shifts, and provide timely emotional support, thereby strengthening their commitment to safeguarding patient rights [50,51]. Furthermore, high-frequency clinical practice in Chinese clinical settings further reinforces their empathy skills. In contrast, lower scores in the leadership dimension revealed deficiencies in developing nursing staff’s capabilities in teamwork, decision-making, and organizational management [52]. From an educational perspective, mainland China’s nursing education system has prioritized clinical skills and foundational nursing knowledge while neglecting systematic leadership development [53,54]. Not only do academic programs lack relevant course offerings, but nurses also receive minimal targeted leadership training or mentorship upon entering clinical settings [54]. This dual disconnection between education and practice, results in Chinese nurses lacking clear awareness of their leadership development potential and struggling to accumulate relevant experience effectively in clinical settings. Furthermore, hierarchical structures within Chinese healthcare systems partially restrict nurses’ initiative in decision-making and management, further hindering the formation and enhancement of their leadership capabilities. Future efforts should prioritize strengthening leadership-related content in nursing education and continuing education, as well as provide chances for nurses to practice in clinical settings, to promote the comprehensive enhancement of nurses’ professional competencies.

T-test results indicate that both nurses’ marital status and whether they obtained OR specialty education or not statistically impacted PPC scores (*p* < 0.05). Married nurses (129.99 ± 25.34) scored markedly higher than unmarried nurses (116.12 ± 26.25). Multiple factors may underline this disparity. On one hand, most married nurses tend to be older and possess more extensive practical experience in ORs, which enhances their clinical adaptability and procedural precision. On the other hand, marital life often entails greater family responsibilities. Within China’s family-oriented sociocultural context, such experiences foster comprehensive competencies in communication, collaboration, emotional regulation, and multitasking. These skills positively influence the development of PPC [55,56]. Nurses who obtained OR specialty education also demonstrated significantly higher PPC scores (127.57 ± 25.76) compared to those without such training (120.06 ± 27.15). This finding emphasizes that hospitals should provide sufficient OR-related specialty education to OR nurses, to foster their systematic knowledge transmission, standardized skill training, and professional competent development. Through specialty education, nurses gain a deeper understanding of perioperative nursing essentials, master the latest clinical guidelines and operational standards, and consequently demonstrate higher professional competence in real-world clinical settings.

ANOVA revealed that PPC scores were statistically significantly influenced by multiple factors (*p* < 0.05). These factors encompassed not only objective indicators such as “age”, “time spent working in the OR (month)”, “professional rank”, “monthly salary”, and “employment type” but also subjective indicators like “self-awareness of current physical condition”. By age group, nurses aged 26–35, 36–45, and ≥46 had significantly higher PPC scores than those aged ≤25. Similarly, nurses aged 36–45 demonstrated statistically significantly higher PPC scores than those aged 26–35. This may be attributed to older nurses having more time spent on clinical practices and specialized education, which fostered greater psychological resilience, better recognition of workplace danger signs, enhanced maturity in communication and teamwork, and stronger professional identity and sense of responsibility [57], resulting in better PPC performances. Regarding time spent working in the OR (month), PPC scores for 13–120 months, 121–240 months, and ≥241 months were significantly higher than those for ≤12 months. Similarly, PPC scores for 121–240 months and ≥241 months were substantially higher than those for 13–120 months. These outcomes may occur because greater experience leads to the automation of repetitive routines, thereby freeing up nurses’ cognitive resources for other demands that require their attention. Additionally, years of practice fostered mastery of OR equipment and resources, alongside internalized proficiency in diverse contingency protocols. Also, the PPC scores of junior registered nurses were significantly lower than those of senior registered nurses, nurses in charge, and associate professor nurses. Similarly, the scores of senior registered nurses were lower than those of nurses in charge. These discrepancies may arise due to nurses in higher-level positions inherently undertaking more advanced responsibilities—such as quality control, systemic risk management, clinical teaching, and departmental decision-making [58]—which foster a broader perspective and deepen their professional understanding. This, in turn, directly elevates their PPC scores. Nurses with monthly incomes between CNY 10,001 and CNY 15,000 and those earning over CNY 15,000 scored significantly higher than those earning CNY 10,000 or less. Higher monthly income correlates with higher PPC scores, reflecting the combined effects of selective recruitment of highly capable individuals and income incentives. Differences in PPC scores also reveal the impact of varying employment types: becoming a tenured nurse requires navigating intense competition and comprehensive competency assessments [59]. This selection mechanism inherently ensures high personnel capability. Furthermore, the security and sense of belonging provided by this system further motivate continuous professional development and the assumption of critical responsibilities—achievements that contract nurses struggle to attain due to job uncertainty. Finally, nurses who self-reported their health status as “good” demonstrated significantly higher PPC than those who rated their health as “general”. A positive self-perception of physical condition ensures sufficient stamina, consistent attendance, thereby accumulating continuous experience [60]. Moreover, evaluating one’s own health status inherently reflects a sense of self-efficacy [61]. The resulting positive psychological state and self-confidence collectively enhances a nurse’s focus, judgment, and stability within the high-pressure surgical environment, ultimately improving their PPC performance.

The results of correlation analysis and multiple linear regression analysis in this study indicate that self-efficacy significantly enhances PPC, while job burnout markedly reduces PPC. Furthermore, a negative correlation exists between self-efficacy and job burnout. Multiple studies support that nurses with higher self-efficacy tend to demonstrate better job performance. For instance, a cross-sectional study of 141 Korean pediatric nurses indicated that intravenous management knowledge and self-efficacy are significant predictors of intravenous nursing practice levels, exhibiting a significant positive correlation [62]. Another cross-sectional survey involving 1137 Swedish and Norwegian nurses revealed that registered nurses with extensive experience demonstrated higher professional competence and self-efficacy compared to those with moderate experience or recent graduates [63]. Concurrently, the extensive literature indicates that job burnout diminishes nurses’ work capacity. A large-scale cross-sectional study conducted in China, covering 105 hospitals and 50,653 female nurses across 15 provinces and 36 cities, found that job burnout positively correlated with sick leave rates and health-related productivity losses [64]. Furthermore, a systematic review (20 studies included) indicated that job burnout is closely associated with reduced nursing safety quality, decreased patient satisfaction, and diminished organizational commitment and work efficiency among nurses [65]. The negative relationship between self-efficacy and work burnout has also been further validated. A cross-sectional study conducted in Taiwan involving 570 nurses found that job burnout was significantly negatively correlated with both self-efficacy and outcome expectancy [66].

When employing structural equation modeling for testing, the findings revealed that self-efficacy played a fully mediating role between job burnout and PPC. Although unexpected, this discovery precisely addresses the research gap highlighted in the Introduction. Specifically, increased levels of individual job burnout are often accompanied by diminished self-efficacy, which in turn exerts a negative influence on PPC. This path relationship also corroborates the conclusions drawn earlier from correlation analysis and multiple linear regression analysis. The existing literature provides strong support for the association among PPC, self-efficacy, and job burnout. Specifically, nurses with high self-efficacy typically demonstrate superior PPC. A study on nursing faculty members in Turkish universities (N = 548) found that the frequency of educational skill utilization was positively associated with the faculty’s general self-efficacy, which then improve their job performance [67]. Another survey conducted in mainland China involving 2970 nurses demonstrated a significant positive correlation between spiritual care competence and self-efficacy (correlation coefficient r = 0.490, *p* < 0.01) [68]. Furthermore, multiple studies confirm a significant negative correlation between self-efficacy and job burnout, positioning self-efficacy as a key protective factor against burnout. A study involving 186 psychiatric nurses examined the impact of job burnout on the relationship between self-efficacy and job performance during the COVID-19 pandemic, and revealed a significant negative correlation between nurses’ self-efficacy and job burnout (r = −0.22, *p* = 0.002) [69]. Simultaneously, job burnout has been demonstrated to negatively impact job competence and self-efficacy. A study conducted in Australia (N = 942) compared changes in multiple psychological and occupational indicators among nursing staff before and after the implementation of an enterprise-wide electronic health record system. Results indicated a significant increase in reported burnout symptoms among nurses post-implementation (95% CI 4.6–4.7%, *p* = 0.036), alongside a marked decline in work engagement (95% CI 49.6–49.9%, *p* < 0.001) [70]. Another study involving nursing students (N = 1445) examining the interrelationships among academic burnout, professional attitudes, academic self-efficacy, and smartphone addiction, revealed that both positive professional attitudes and higher academic self-efficacy were negatively correlated with academic burnout [71]. Furthermore, a study of Polish nurses (N = 405) which examined how work environments and related factors influence healthcare workers’ self-efficacy indicated that lower self-efficacy levels were associated with more burnout symptoms, revealing a stable negative correlation with a correlation coefficient r∈[−0.19, −0.17] [72]. Collectively, this evidence forms a mutually reinforcing logical framework, solidifying the theoretical foundation for the variable relationships examined in this study.

## 5. Limitations

A key limitation of this study stems from its sampling technique. The stratified convenience sampling likely lacks the representativeness of a true probability sample of all Chinese OR nurses. Thus, the measured reliability is specific to the included cohort, and any application of these findings to the general population must be undertaken prudently. Additionally, due to the cross-sectional and self-reported nature of this study, the results may be susceptible to common method bias. To mitigate this risk, we emphasized respondent anonymity and confidentiality in the research design to minimize socially desirable responses.

## 6. Conclusions

This is the first study in China to demonstrate self-efficacy as a full mediator between burnout and self-efficacy among OR nurses. Therefore, enhancing self-efficacy is key to effectively strengthen PPC and alleviate job burnout among these nurses. To enhance OR nurses’ self-efficacy, systematic and comprehensive measures are required. These include, but are not limited to, providing phased, scenario-based skill training to OR nurses at the organizational level; establishing mentorship programs and constructive feedback mechanisms at the team level to foster a supportive work environment; promoting nurses’ sense of success through empowering participation in decision-making and role modeling; and prioritizing mental health support to fundamentally strengthen their professional confidence and competence. Future research should design interventions to enhance operating room nurses’ self-efficacy, reducing burnout, and strengthening PPC through randomized controlled trials.

## Figures and Tables

**Table 1:** Demographic information of included participants and results of *t*-test and ANOVA test.

Variable	N (%)	M ± SD	*t*/*F* Value	*p* Value	Further Pairwise Comparisons	*p* Value
**Gender**			0.16	0.874		
Male	105 (16.75)	124.92 ± 27.28				
Female	522 (83.25)	124.47 ± 26.44				
**Marital status**			−6.60	<0.001 ***		
Unmarried	246 (39.23)	116.12 ± 26.25				
Married	381 (60.77)	129.99 ± 25.34				
**Obtained OR specialty education**			3.50	<0.001 ***		
Yes	375 (59.81)	127.57 ± 25.76				
No	252 (40.19)	120.06 ± 27.15				
**Age**			25.02	<0.001 ***	(1) < (2)	<0.001 ***
≤25 (1)	133 (21.21)	110.62 ± 22.17			(1) < (3)	<0.001 ***
26~35 (2)	328 (52.31)	124.44 ± 27.05			(1) < (4)	<0.001 ***
36~45 (3)	124 (19.78)	136.56 ± 24.65			(2) < (3)	<0.001 ***
≥46 (4)	42 (6.70)	134.02 ± 19.26				
**Time spent working in the OR (month)**			32.47	<0.001 ***	(1) < (2)	<0.001 ***
≤12 (1)	107 (17.07)	106.55 ± 22.82			(1) < (3)	<0.001 ***
13~120 (2)	302 (48.17)	123.27 ± 25.79			(1) < (4)	<0.001 ***
121~240 (3)	165 (26.32)	135.39 ± 24.78			(2) < (3)	<0.001 ***
≥241 (4)	53 (8.45)	134.43 ± 22.28			(2) < (4)	0.015 *
**Highest education**			0.62	0.527		
Junior college or below (1)	123 (19.62)	123.95 ± 26.17				
Bachelor (2)	496 (79.11)	124.86 ± 26.56				
Graduate or above (3)	8 (1.28)	114.63 ± 33.74				
**Professional rank**			18.68	<0.001 ***	(1) < (2)	<0.001 ***
Junior registered nurse (1)	124 (19.78)	111.28 ± 24.00			(1) < (3)	<0.001 ***
Senior registered nurse (2)	286 (45.61)	124.08 ± 25.99			(1) < (4)	0.001 **
Nurse in charge (3)	190 (30.30)	132.78 ± 25.82			(2) < (3)	0.002 **
Associate professor nurses (4)	27 (4.31)	132.48 ± 24.65				
**Monthly income**			7.39	<0.001 ***	(1) < (2)	0.006 **
≤10,000 (1)	439 (70.02)	122.07 ± 27.10			(1) < (3)	0.027 *
10,001~15,000 (2)	168 (26.79)	129.44 ± 24.53				
>15,000 (3)	20 (3.19)	137.80 ± 22.08				
**Employment type**			4.08	0.017 *	(1) > (2)	0.013 *
Tenured nurse (1)	91 (14.51)	131.86 ± 23.89				
Contract nurse (2)	528 (84.21)	123.29 ± 26.74				
Others (3)	8 (1.28)	124.50 ± 33.27				
**Self-awareness of current physical condition**			13.29	<0.001 ***	(2) < (3)	<0.001 ***
Poor (1)	22 (3.51)	130.23 ± 24.33				
General (2)	488 (77.83)	121.72 ± 26.27				
Good (3)	117 (18.66)	135.26 ± 25.44				

“N” refers to frequency; “%” referrers to percentage; “M ± SD” refers to mean ± standard deviation; “t/F Value” refers to the t value of the *t*-test or F value of the ANOVA test; “<” and “>” refer to statistically less than or more than; “***” means *p* < 0.001, “**” means *p* < 0.01, “*” means *p* < 0.05.

**Table 2:** Mediation analysis.

Mediation Path	Estimate	CR.	*p* Value	Total Effect	Indirect Effect	Direct Effect
Self-efficacy ← Job burnout	−0.28	−4.82	<0.001 ***			
PPC ← Self-efficacy	0.64	10.56	<0.001 ***			
PPC ← Job burnout	−0.07	−1.76	0.078	−0.25	−0.18	−0.07
95% CI (bootstrap method)				[−0.314, −0.08]	[−0.257, −0.099]	[−0.159, 0.005]
*p*				0.013 *	0.020 *	0.062

“Mediation Path” refers to path effects/variable relationships, representing the path relationships defined in the model; “Estimate” denotes the estimated path coefficient, which is a standardized or unstandardized regression coefficient (β value), indicating the magnitude and direction of the independent variable’s influence on the dependent variable; “CR.” denotes the Critical Ratio/t-value, a statistic used to test whether coefficients are significant; “*p* Value” refers to the *p* value for effects; “Total Effect” is the overall impact of the independent variable on the dependent variable (Direct Effect + Indirect Effect); “Indirect Effect” is the mediating effect, i.e., the effect transmitted through the mediating variable; “Direct Effect” is the direct impact of the independent variable on the dependent variable without mediation by the intervening variable; 95% CI refers to the bootstrap confidence interval; “***” means *p* < 0.001; “*”means *p* < 0.05.

## Data Availability

The original contributions presented in this study are included in the article. Further inquiries can be directed to the corresponding author. The data presented in this study are available on request from the corresponding author due to ethical concerns.

## Abbreviations

The following abbreviations are used in this manuscript:

PPC	Perceived Perioperative Competence
OR	Operating Room
C-PPCS-R34	34-item Chinese Perceived Perioperative Competence Scale-Revised
ANOVA	Analysis of Variance
PPCS-R	Perceived Perioperative Competence Scale-Revised
GSES-C	Chinese version General Self-Efficacy Scale
MBI-HSS	Maslach Burnout Inventory-Human Services Survey
SD	Standard Deviation
df	Degree of Freedom
RMSEA	Root Mean Square Error of Approximation
SRMR	Standardized Residual Mean Root
GFI	Goodness-of-Fit Index
NFI	Normed Fit Index
RFI	Robust Fitting Index
IFI	Incremental Fit Index
TLI	Tucker–Lewis Index
CFI	Comparative Fit Index

## Appendix A

**Table A1:** Results of correlation analysis.

Variable	PPC (*r*/*p* Value)	Self-Efficacy	Job Burnout
PPC	1.000		
Self-efficacy	0.604 (<0.001 ***)	1.000	
Job burnout	−0.357 (<0.001 ***)	−0.378 (<0.001 ***)	1.000

“*r*” refers to correlation coefficient; “*p*” refers to *p* value; “***” means *p* < 0.001.

## Appendix B

**Table A2:** Results of multiple linear regression analysis.

Variable	*B* Coefficient	*β* Coefficient	*t* Value	*p* Value	*F* Value	Adjusted R-Squared
Constants	62.85	-	12.07	<0.001 ***	194.23	0.38
Self-efficacy	2.46	0.55	16.10	<0.001 ***		
Job burnout	−0.21	−0.15	−4.44	<0.001 ***		

“*B* Coefficient” refers to unstandardized regression coefficient; “*β* Coefficient” refers to standardized regression coefficient; “*t* Value” refers to t-statistic; “*p* Value” refers to *p* value; “*F* Value” refers to F-statistic; “R” refers to multiple correlation coefficient; “***” means *p* < 0.001; in this model, R = 0.619, R^2^ = 0.384, *p* < 0.001.
